# Case of Fever

**Published:** 1856-12

**Authors:** Thos. S. Denny

**Affiliations:** Atlanta, Ga.


### ARTICLE IV.

*Case of Fever.* By Thos. S. Denny, M. D., Atlanta, Ga.

The great interest evinced in the subject before our last meeting, induces me to present a statement of the following case of Fever. I am desirous that each member of the Profession should form his own opinion of the nature of the Fever; my own is, that it was originally a case of Catarrhal Fever, having a very strong tendency to the Typhoid type. The patient is of a delicate constitution, about —— years of age, and never had any sickness before, except slight attacks of Catarrh. His constitution is lymphatic—predisposed to phthisis pulmonalis—male parent died of it. He resided in what I consider an unhealthy location, in the neighborhood of one of the filthiest branches in the city.

I was called on Saturday, 6th September last, at dark; found patient with very dry and hot skin, pulse, 85, dry and short cough; very slight uneasiness in chest, and no pain in any other part of the body; tongue furred, white. Prescribed pulv. ipecac, c. grs. x. hyd. prot. chlod. gr. i.

7th, 8½, A. M.—Symptoms much the same as yesterday· Repeat pulv. and the following mixture, syr. scillæ, tinct. op., camph., and vin. antim.

8th.—Repeat pulv. and continue mixture.

9th.—Fever very hot all night; abated this morning. Ordered sulph. quinine, 3 gr. doses, and hyd. prot. chlorid. gr. i. to each dose: one every two or three hours. Continue mixt.

10th.—Fever much diminished yesterday; after quinine, repeat pulv.

11th. Fever increased at evening. Continue quinine and mixture.

12th.—Fever much diminished; continue quinine and mixture. Patient very sleepless last night; ⅛ gr. acet. morph. at bed-time.

13th.—Morph. produced very happy effects; slept well and feels much better—no fever—continue quinine and morph.

14th.—Very much improved; discontinue quinine; give morphine at bed-time.

15th.—Improving; bowels have, all along, been disposed to be natural—rather inactive to-day—ordered charcoal, ℥jj. in milk, as aperient, and ⅛ gr. morph. at bed-time.

16th.—Tongue, which has been all along coated, white at first, then brownish, now begins to clear. Morph. ⅛ gr.

17th.—Patient sitting up; feels stronger; tongue cleaning. Morphine, ⅛ gr.

18th.—Patient slept until 2 o'clock this morning, without morph.; skin rather warmer than yesterday; appetite increasing; morph., 1-10 gr.

19th.—Slept until 3 o'clock, with morph., ⅛ gr.; skin perfectly natural; tongue, ditto. Nothing but debility remaining; morph., 1-10 grain.

20th.—Morphine, 1-10 grain.

22d.—Attempted to sleep without powder last night, and woke three times in cold perspiration, and had pains over the body. Pulv. ipecac, c. and cal., gr. ii. in pulv. no. ii.

23d.—Same.

24th.—Repeat pulv.; relieved.

25th.—Omit calomel.

26th.—Pulv.

27th.— do.

30th.—Nit. mur. acid, ℥j. gtt. x *ter in die.*

The patient continued to improve, but for imprudences in diet, and unnecessary exposure, and is now at his usual avocations. Several nights the patient's mind wandered considerably, but he was perfectly rational during the day.

